# SOCIAL HEALTH IN PEOPLE WITH DEMENTIA AND THEIR CARERS. PRELIMINARY FINDINGS FROM THE IDEAL PROGRAM

**DOI:** 10.1093/geroni/igad104.1431

**Published:** 2023-12-21

**Authors:** Christina Victor, Isla Rippon

**Affiliations:** Brunel University-London, Uxbridge, England, United Kingdom; Brunel University London, Uxbridge, England, United Kingdom

## Abstract

WHO defines health as a state of complete physical, mental, and social wellbeing. There is an extensive literature examining physical and mental health in later life and, typically, measures of healthy ageing focus upon physical and/or mental health. In the IDEAL dementia research programme we investigated social health outcomes because of their importance in understanding the experience of ‘living well’ for people with dementia. Using data from the IDEAL cohort study, we examined the prevalence and predictors of social isolation in 1,052 people with mild-to-moderate dementia and 928 caregivers who were followed up at 12 and 24 months. Social isolation was assessed using the six-item Lubben Social Network Scale where a score of less than 12 suggests participants are at risk of social isolation. Linear mixed models showed that both people with dementia and caregivers experienced increased social isolation across the two-year period. At baseline 29% of people with dementia were categorised as being socially isolated compared with 14% of caregivers. For both people with dementia and caregivers, loneliness and depression were associated with greater social isolation whilst increased cultural engagement mitigated the impact of social isolation. For people with dementia cognition, the number of green and blue spaces nearby and higher trust in the local community were also important factors. Interventions aimed at increasing cultural engagement and interaction with blue and green spaces may go some way to reducing social isolation.

